# Mechanisms Underlying Apoptosis-Inducing Effects of Kaempferol in HT-29 Human Colon Cancer Cells

**DOI:** 10.3390/ijms15022722

**Published:** 2014-02-17

**Authors:** Hyun Sook Lee, Han Jin Cho, Rina Yu, Ki Won Lee, Hyang Sook Chun, Jung Han Yoon Park

**Affiliations:** 1 Department of Food Science and Nutrition, Dongseo University, 47 Jurye-ro, Sasang-gu, Busan 617-716, Korea; E-Mail: hyunlee@gdsu.dongseo.ac.kr; 2 Department of Food and Nutrition, Hallym University, Chuncheon 200-702, Korea; E-Mails: hanjini@hallym.ac.kr; 3 Department of Food Science and Nutrition, University of Ulsan, Ulsan 680-749, Korea; E-Mail: rinayu@ulsan.ac.kr; 4 Advanced Institutes of Convergence Technology, Seoul National University, Suwon, Gyonggi-do 443-270, Korea; E-Mail: kiwon@snu.ac.kr; 5 WCU Biomodulation Major, Department of Agricultural Biotechnology and Center for Food and Bioconvergence, Seoul National University, Seoul 151-921, Korea; 6 Food Science and Technology, Chung-Ang University, An-Sung 456-756, Korea; E-Mail: hschun@cau.ac.kr

**Keywords:** kaempferol, colon cancer, apoptosis, mitochondrial pathway, cell-death receptor pathway

## Abstract

We previously noted that kaempferol, a flavonol present in vegetables and fruits, reduced cell cycle progression of HT-29 cells. To examine whether kaempferol induces apoptosis of HT-29 cells and to explore the underlying molecular mechanisms, cells were treated with various concentrations (0–60 μmol/L) of kaempferol and analyzed by Hoechst staining, Annexin V staining, JC-1 labeling of the mitochondria, immunoprecipitation, *in vitro* kinase assays, Western blot analyses, and caspase-8 assays. Kaempferol increased chromatin condensation, DNA fragmentation and the number of early apoptotic cells in HT-29 cells in a dose-dependent manner. In addition, kaempferol increased the levels of cleaved caspase-9, caspase-3 and caspase-7 as well as those of cleaved poly (ADP-ribose) polymerase. Moreover, it increased mitochondrial membrane permeability and cytosolic cytochrome c concentrations. Further, kaempferol decreased the levels of Bcl-xL proteins, but increased those of Bik. It also induced a reduction in Akt activation and Akt activity and an increase in mitochondrial Bad. Additionally, kaempferol increased the levels of membrane-bound FAS ligand, decreased those of uncleaved caspase-8 and intact Bid and increased caspase-8 activity. These results indicate that kaempferol induces the apoptosis of HT-29 cells via events associated with the activation of cell surface death receptors and the mitochondrial pathway.

## 1. Introduction

Colon cancer is one of the principal causes of cancer-associated deaths in the Western world; in Asia, such incidence is progressively increasing, which may be due to changes in lifestyle, particularly changes in dietary habits [1–3]. Recently, in order to prevent colon cancer, efforts have been made to identify naturally occurring dietary compounds as well as to use them as cancer chemopreventive agents.

**Figure 1.**
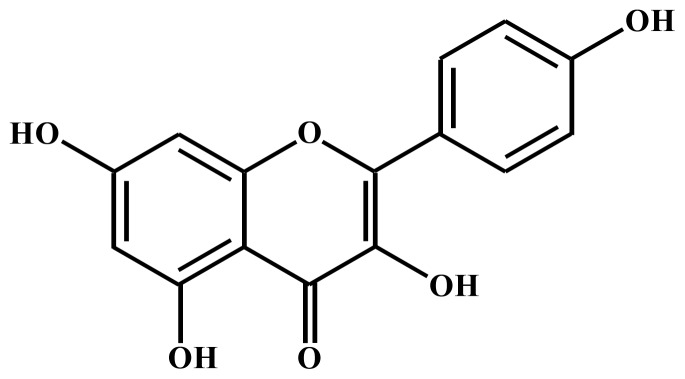
Structure of kaempferol.

Flavonoids, a group of polyphenolic compounds, exist ubiquitously in vegetables and fruits. They are classified as flavonols, flavones, catechins, anthocyanidins and isoflavonoids, according to their heterocyclic rings [4]. Kaempferol (3,4′,5,7-tetrahydroxyflavone, Figure 1), a flavonol, is a natural polyphenol compound present in vegetables and fruits, including onions, apples and green and black tea. A number of epidemiological studies have documented that a high intake of kaempferol is associated with decreased risk of colon cancer [5], ovarian cancer [6], pancreatic cancer [7] and gastric cancer [8]. Additionally, several previous studies have reported that kaempferol has anti-proliferation activities and/or induces apoptosis in various human cancer cell lines, including lung cancer [9,10], esophageal cancer [11], prostate cancer [12] and colon cancer cells [13,14].

On the molecular level, it has been reported that kaempferol induces the apoptosis of cancer cells via a wide variety of mechanisms. For example, kaempferol-induced cell death is dependent upon the activation of MEK-MAPK (mitogen-activated protein kinase) in A549 lung cancer cells [10]. Kaempferol induces the caspase-3-dependent apoptosis in oral cavity cancer cells [15]; sensitizes SW480 colon cancer cells to tumor necrosis factor-related apoptosis-inducing ligand (TRAIL)-induced apoptosis [13]; and induces apoptosis and a G2/M cell cycle arrest, which are associated with increases in the expression and phosphorylation of the tumor suppressor protein p53 in human breast cancer cells [16]. In human HCT116 colon cancer cells, which contain the wild type *p53* gene [17], kaempferol induces p53-dependent growth inhibition and apoptosis [18]. Despite several previous studies which have reported that kaempferol demonstrates anti-cancer activity in various human cancer cell lines [9–16,18], detailed molecular mechanisms responsible for their effects on colon cancer cells still remain largely unknown.

We recently reported that kaempferol induces G1 and G2/M cell cycle arrest of HT-29 cells by inhibiting the activity of cyclin-dependent kinase (CDK)2, CDK4, and Cdc2 [19]. p53, also known as protein 53, is a tumor suppressor protein that in humans is encoded by the *TP53* gene [20]. Further, *TP53* is one of the most frequently mutated genes in human cancers, including colon cancer (reviewed in [21]). Therefore, the purpose of the present study is to investigate whether kaempferol induces the apoptosis of HT-29 human colon cancer cells that contain the mutant *p53* gene [17] as well as to elucidate the molecular mechanisms underlying this effect. The results of our experiments indicate that kaempferol exerts distinct apoptotic effects via the modification of Bcl-2 family proteins, leading to increases in the permeability of mitochondrial membranes and caspase-9-mediated caspase activation. Additionally, kaempferol-induced apoptosis of HT-29 cells is also dependent on the extrinsic pathway modulated by the FAS ligand (FAS-L)/receptor pathways involving the activation of caspase-8. Furthermore, kaempferol inhibits Akt activation as well as kinase activity, which leads to an increase in Bad translocation to the mitochondria.

## 2. Results and Discussion

### 2.1. Kaempferol Induces Apoptosis in HT-29 Cells

Over the past several decades, many effective anti-cancer drugs have been developed and are currently being used to treat cancer patients. However, most of these drugs still have serious side effects; therefore, natural compounds are receiving more attention today for their ability to prevent and/or delay cancer development. In humans, the major sources of kaempferol intake are tea, onions and apples [4]. According to epidemiological studies, the proportions of dietary flavonol/flavone intakes of total flavonoids in American men and women are 20% and 22%, respectively [22]. It has been reported that the intake of kaempferol accounts for 35% of the total flavonoid intake in Japanese women [23]. The results from studies utilizing a variety of different type of cancer cells indicate that kaempferol has an anti-cancer potential [9,11,12,14,24]. In colon cancer cells, kaempferol increases the sensitivities of SW480 colon cancer cells to TRAIL [13] and induces p53-dependent growth inhibition and apoptosis in HCT116 cells [18]. These results reveal that kaempferol has the potential to be used as an agent to prevent and/or treat colon cancer.

**Figure 2.**
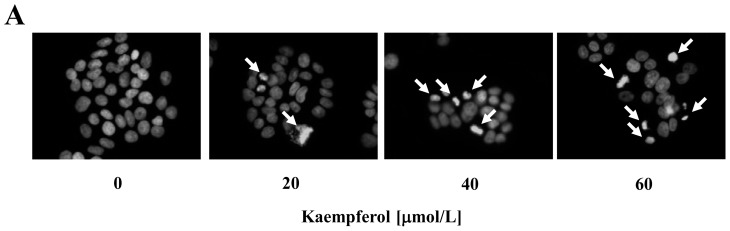

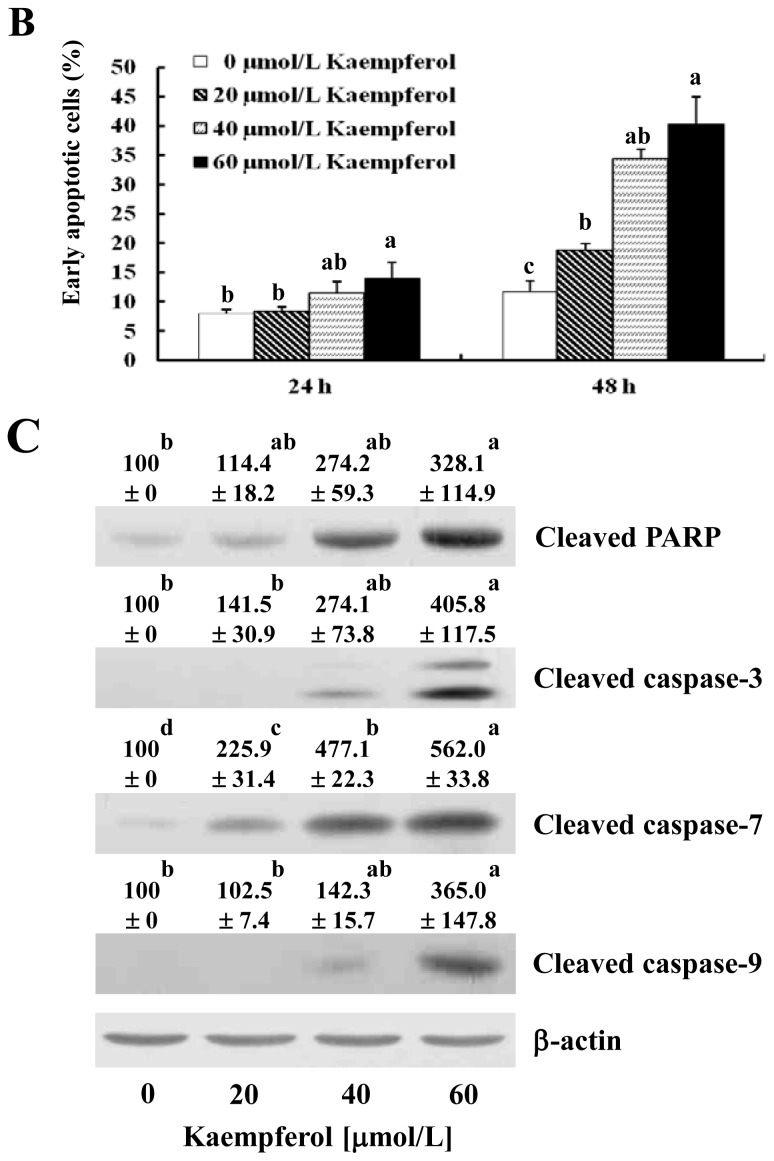
Effect of kaempferol on apoptosis of HT-29 cells. (**A**) Cells were plated in 4-well chamber slides and treated with various concentrations of kaempferol for 48 h. Cells were fixed and stained with Hoechst 33258. Arrows indicate apoptotic cells; (**B**) Cells were plated in 24-well plates and treated with various concentrations of kaempferol for 24 or 48 h. Cells were trypsinized, loaded with Annexin V and 7-amino-actinomycin D (7-AAD), and analyzed by flow cytometry. The percentages of early apoptotic cells (Annexin V^+^/7-AAD^−^ cells) were calculated. Each bar represents the mean ± SEM (*n* = 6); (**C**) Cells were plated in 100-mm dish and treated with various concentrations of kaempferol for 48 h. Total cell lysates were analyzed via Western blotting with antibodies against the indicated antibodies. The relative abundance of each band was quantified and the control levels (0 μmol/L kaempferol) were set at 100%. The adjusted mean ± SEM (*n* = 3) of each band is shown above each blot. Means without the same letter (a, b, c or d) are significantly different (*p* < 0.05).

**Figure 3.**
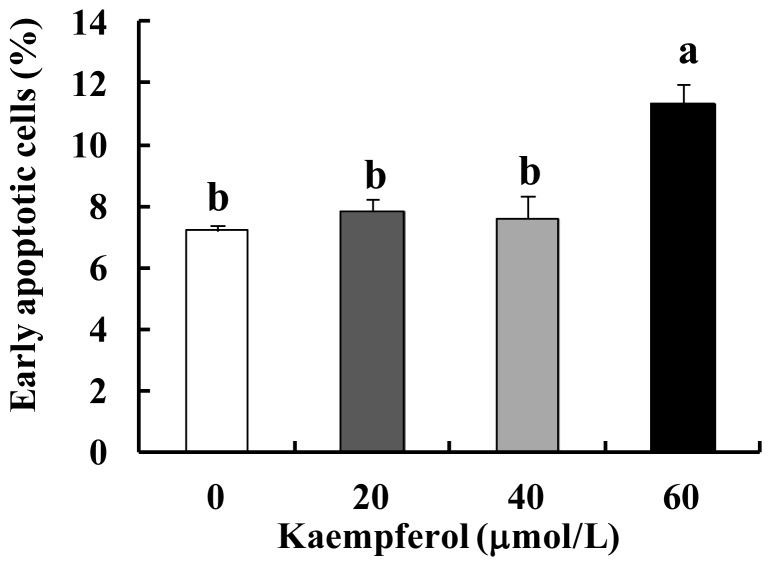
Effect of kaempferol on apoptosis of SW480 cells. Cells were plated in 24-well plates and treated with various concentrations of kaempferol for 48 h. Cells were trypsinized, loaded with Annexin V and 7-AAD, and analyzed by flow cytometry. The percentages of early apoptotic cells were calculated. Each bar represents the mean ± SEM (*n* = 3). Means without the same letter (a or b) are significantly different (*p* < 0.05).

Our previous study demonstrated that kaempferol inhibits cell proliferation and induces cell cycle arrest in HT-29 human colon cancer cells [19]. Apoptosis is an important event leading to programmed cell death that is also essential for normal physiology, such as development and maintenance of the organism. Cancer cells adopt various strategies to override apoptosis [25]. Therefore, in order to effectively prevent or treat cancer, it is desirable to find agents that have the abilities to selectively kill cancer cells and simultaneously, to protect normal cells. In the present study, to investigate whether kaempferol induces apoptosis of HT-29 cells, HT-29 colon cancer cells were treated with 0, 20, 40 and 60 μmol/L of kaempferol for 24 or 48 h. Hoechst staining results revealed that kaempferol induced the chromatin condensation and DNA fragmentation in HT-29 cells, which are important characteristics of apoptotic cells (Figure 2A). Additionally, Annexin V staining results demonstrated that kaempferol treatment increased the percentage of early apoptotic cells in a dose- and time-dependent manner (Figure 2B). These data clearly indicate that kaempferol induces apoptosis in HT-29 cells. In addition to HT-29 cells, we also examined whether kaempferol induces apoptosis of SW480 human colon cancer cells containing a mutant p53. Annexin V staining results revealed kaempferol significantly (*p* < 0.05) induced apoptosis of SW480 cells at a concentration of 60 μmol/L (Figure 3). When the same concentrations of kaempferol were added to the culture of IEC-6 cells, a normal intestinal epithelial cell line, the viability of cells did not change significantly (data not shown).

### 2.2. Kaempferol Activates the Caspase Cascade in HT-29 Cells

Caspases, a family of cysteine-aspartic proteases, play an essential role in apoptosis. Caspases are first synthesized as inactive pro-caspases and regulated at a post-translational level. Activated caspases eventually lead to apoptotic cell dismantling (reviewed in [26]). The initiator caspase, caspase-9 cleaves and activates caspase-3 and caspase-7 which are responsible for the execution of apoptosis. To determine whether kaempferol-induced apoptosis is associated with the activation of the caspase cascade, we measured the activation of caspase-9, caspase-3 and caspase-7 by detecting the cleaved forms in each caspase. We also measured the levels of cleaved poly (ADP-ribose) polymerase (PARP), a family of proteins involved in a variety of cellular processes including DNA repair and programmed cell death, and also a downstream target of caspase-3 [27–29]. The results from the Western blot analysis revealed that the levels of the active (cleaved) forms of these caspases and cleaved-PARP were increased in a dose-dependent manner in kaempferol-treated HT-29 cells (Figure 2C).

### 2.3. Kaempferol Modulates the Levels and Localization of Bcl-2 Family Proteins in HT-29 Cells

**Figure 4.**
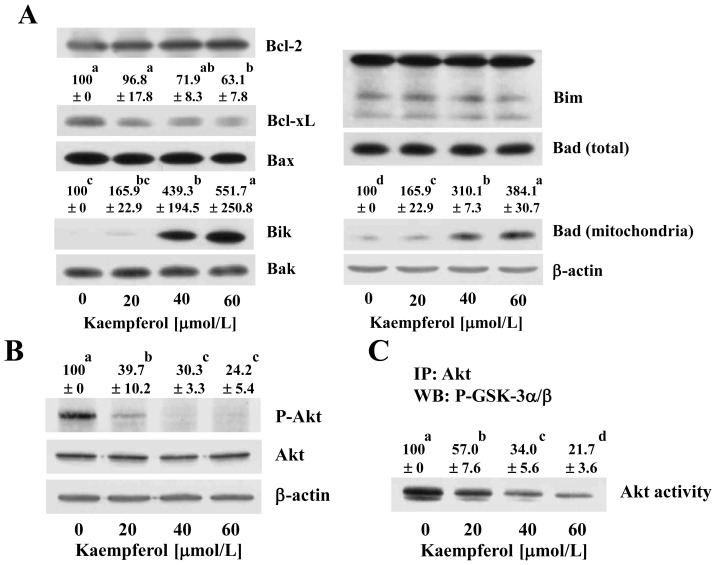

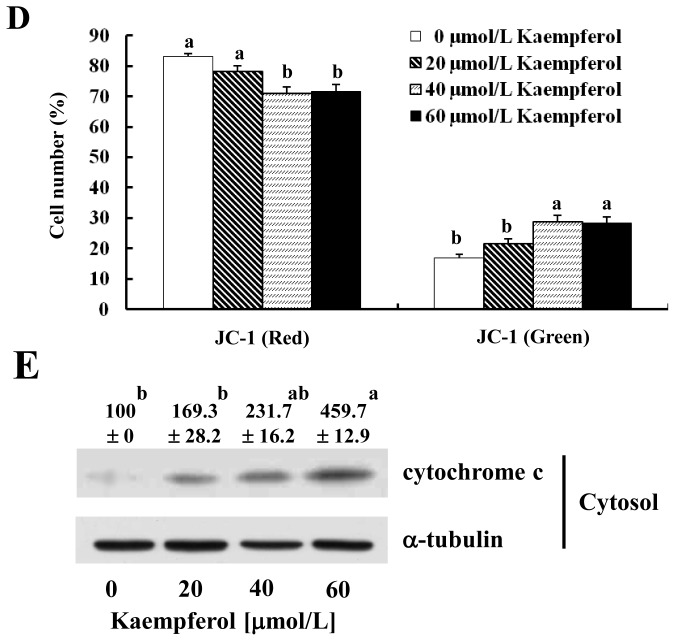
Kaempferol induces changes in the levels and localization of Bcl-2 family proteins. Cells were treated with various concentrations of kaempferol for 48 h. (**A**,**B**,**E**) Total cell lysates and subcellular fractions were prepared and analyzed via Western blotting with antibodies against the indicated antibodies; (**C**) Total cell lysates were incubated with immobilized Akt antibody, and the immunoprecipitated proteins were incubated with GSK-3α/β (a substrate) and ATP. Each sample was analyzed via Western blotting with anti-P-GSK-3α/β antibody; (**A**,**B**,**C**,**E**) The relative abundance of each band was quantified and the control levels (0 μmol/L kaempferol) were set at 100%. The adjusted mean ± SEM (*n* = 3) of each band is shown above each blot; (**D**) Cells were trypsinized, loaded with JC-1 and analyzed by flow cytometry. Each bar represents the mean ± SEM (*n* = 6). Means without the same letter (a, b, c or d) are significantly different (*p* < 0.05).

Activation of apoptosis is initiated by two main pathways, the intrinsic and/or extrinsic pathway [30]. The mitochondrion is an essential mediator of intrinsic apoptosis. The Bcl-2 family of proteins, located on the outer mitochondrial membrane, is important in modulating apoptosis by altering the mitochondrial permeability to the apoptotic mediators. The ratio of pro-apoptotic (Bax, Bid, Bik) and anti-apoptotic (Bcl-2, Bcl-xL) proteins determines the relative amount of cytochrome c between the cytosol and mitochondria. Because apoptosis in mammalian cells has been shown to be regulated by Bax, Bcl-xL, Bad and Bcl-2 [31], we next determined whether kaempferol-induced apoptosis in HT-29 cells is also associated with the modulation of these proteins. Western blotting results indicated that kaempferol treatment decreased the expression of Bcl-xL and increased the expression of Bik; however, it did not change the levels of anti-apoptotic Bcl-2 or pro-apoptotic Bak or Bax. Kaempferol treatment had no effect on the levels of total Bad, whereas kaempferol increased the levels of Bad in the mitochondria (Figure 4A).

Akt, a phsophoinositide-3-OH kinase activated protein kinase, has direct effects on the apoptosis machinery and furthermore, promotes cell survival (Reviewed in [32]). Previously, we have demonstrated that insulin-like growth factor (IGF)-I and heregulin stimulate cell growth and activate Akt in HT-29 cells. We also noted that HT-29 cells produce IGF-II and heregulin, which stimulate Akt activation via autocrine mechanisms thereby increasing cell survival [33,34]. Kaempferol treatment reduced the levels of phospho-Akt without causing changes in the total levels of Akt (Figure 4B). In order to examine whether kaempferol inhibits Akt activity in HT-29 cells, Akt was immunoprecipitated from total cell lysates, and kinase activity was estimated using GSK-3α/β as a substrate. Akt activity was reduced in HT-29 cells treated with kaempferol in a dose-dependent manner (Figure 4C). Bad is a substrate of Akt, and after being phosphorylated by Akt, Bad is sequestrated with 14-3-3 protein in the cytosol (reviewed in [32]). In the mitochondria, Bad interacts with anti-apoptotic Bcl-2 family proteins, such as Bcl-xL, which allows the multidomain proapoptotic Bcl-2 proteins, such as Bax and Bak, to aggregate and increase the permeability of mitochondrial membranes [35,36]. The present results showing a decrease in activated Akt and an increase in mitochondrial Bad in kaempferol-treated cells indicate that kaempferol-induced apoptosis of HT-29 cells is mediated, at least in part, by inactivation of the Akt signaling pathway. Reduced Akt activity allows Bad to translocate to the mitochondria in kaempferol-treated cells, thereby leading to the stimulation of mitochondrial membrane depolarization.

### 2.4. Kaempferol Induces Depolarization of the Mitochondria and the Release of Cytochrome C from the Mitochondria in HT-29 Cells

Changes in mitochondrial outer membrane permeability are critical to the progression of the intrinsic apoptosis pathway. Due to the changes in permeability of the mitochondrial membrane, cytochrome c, one of the pro-apoptotic molecules, is released from the mitochondria in order to trigger caspase-9 activation, leading to caspase-3 activation (reviewed in [37]). In order to determine whether kaempferol-induced apoptosis was mediated by the mitochondrial-dependent pathway, we used JC-1 as a mitochondrial depolarization indicator due to its established ability to detect changes in the mitochondrial membrane potential of living cells. As shown in Figure 4D, kaempferol decreased in the red/green fluorescence intensity ratio, indicating that kaempferol increased mitochondrial membrane permeability. Western blot analysis of the cytosolic fraction revealed that kaempferol caused a dose-dependent increase of cytochrome c in the cytosol (Figure 4E). Together, our results indicate that kaempferol modulates the expression and localization of the Bcl-2 family, which leads to an increase in the permeability of mitochondrial membranes and the subsequent release of cytochrome c into cytosol. This release of cytochrome c, in turn, activates caspase-9 in the cytosol.

### 2.5. Kaempferol Induces Changes in the Levels of Proteins Involved in the Regulation of the Extrinsic Apoptotic Pathway in HT-29 Cells

The activation of apoptosis via the extrinsic pathway occurs with the binding of apoptotic ligands to death receptor (DR)s, such as FAS, TNFR1, DR3, TRAIL-receptor (TRAIL-R)1 or DR4, TRAIL-R2 or DR5 and DR6. These death receptors have an intracellular domain that functions as a protein-binding module. Following the recruitment and signaling of various adaptor molecules, the cleavage and activation of the initiator enzymes caspase-8 and -10 occur. This, in turn, leads to the activation of executioner enzymes caspase-3, -6, and -7, ending with DNA fragmentation (reviewed in [38]).

**Figure 5.**
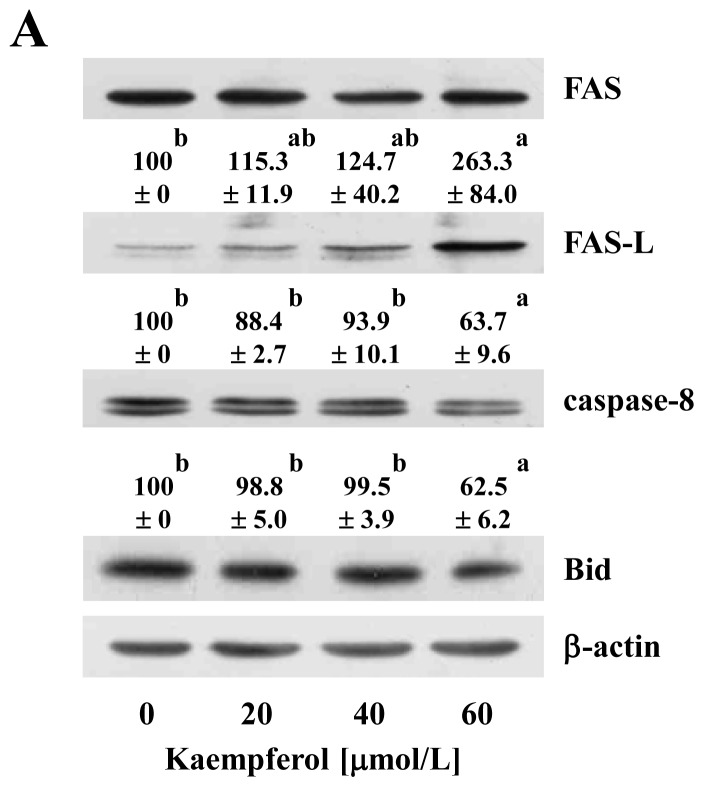

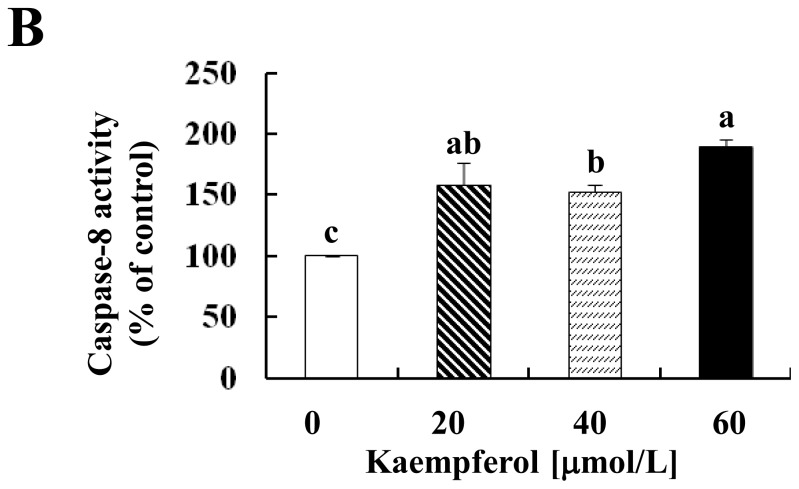
Effect of kaempferol on extrinsic apoptotic pathway. Cells were treated with various concentrations of kaempferol for 48 h. (**A**) Total cell lysates were prepared and analyzed via Western blotting with antibodies against the indicated antibodies. The relative abundance of each band was quantified and the control levels (0 μmol/L kaempferol) were set at 100%. The adjusted mean ± SEM (*n* = 3) of each band is shown above each blot; (**B**) Caspase-8 activity was measured using Caspase-8 colorimetric assay kit in accordance with the manufacturer’s instructions (Clontech, Mountain View, CA, USA). Each bar represents the mean ± SEM (*n* = 4). Means without the same letter (a, b or c) are significantly different (*p* < 0.05).

**Figure 6.**
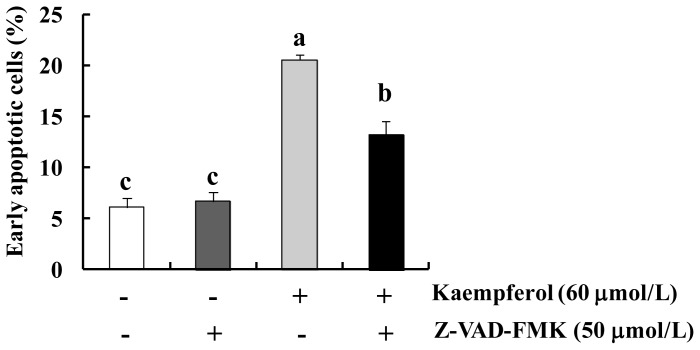
The caspase inhibitor Z-VAD-FMK attenuates kaempferol-induced apoptosis in HT-29 cells. Cells were plated in 24-well plates and treated with 50 μmol/L Z-VAD-FMK for 1 h. Cells were then treated with 0 or 60 μmol/L kaempferol with or without 50 μmol/L Z-VAD-FMK for 48 h. Cells were trypsinized, loaded with Annexin V and 7-AAD, and analyzed by flow cytometry. The percentages of early apoptotic cells were calculated. Each bar represents the mean ± SEM (*n* = 3). Means without the same letter (a, b or c) are significantly different (*p* < 0.05).

We also explored whether kaempferol induces changes in the extrinsic apoptotic pathway. As shown in Figure 5A, kaempferol induced the expression of FAS ligand (FAS-L), but did not alter the expression of FAS. Kaempferol also decreased the levels of uncleaved caspase-8 and Bid. Additionally, kaempferol increased the activity of caspase-8 in HT-29 cells (Figure 5B). Bid, a pro-apoptotic member of the Bcl-2 family containing a BH3 domain, is a specific substrate of caspase-8. Uncleaved Bid is localized in the cytosol as an inactive precursor. When Bid is cleaved by caspase-8, the truncated Bid (tBid) translocates to the mitochondria and thus conveys extrinsic apoptotic signals from the cytoplasm to the mitochondria (reviewed in [39]). Together, the present results indicate that kaempferol activates the FAS-L/FAS, which recruits and activates caspase-8. The activated caspase-8 cleaves Bid and tBid moves to the mitochondria and contributes to the kaempferol-induced activation of the intrinsic apoptotic pathway. However, Li *et al.* [18] demonstrated that kaempferol has no influence on the cleavage of caspase-8 in human HCT116 colon cancer cells, which has the wild type *p53* [17]. The present study did not determine as to why this difference exists between the two cells. Finally, we examined whether the caspase inhibitor Z-VAD-FMK attenuates kaempferol-induced apoptosis of HT-29 cells. The pan-caspase inhibitor significantly blocked the apoptosis induced by kaempferol treatment, indicating that kaempferol induces apoptosis in a caspase-dependent manner (Figure 6).

**Figure 7.**
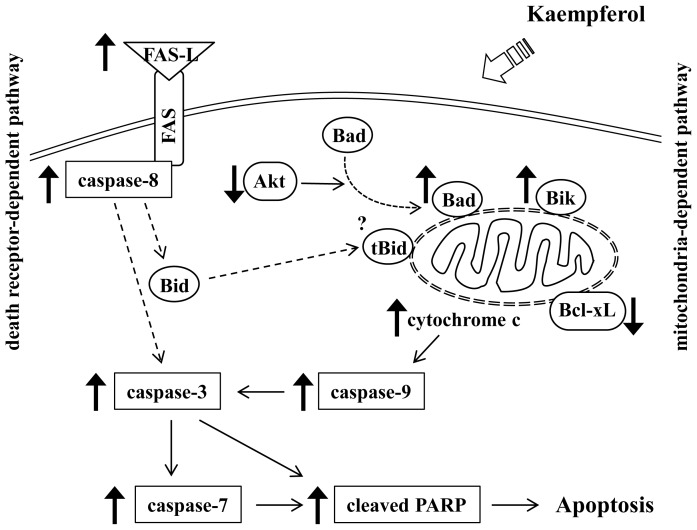
Proposed pathways for kaempferol’s effect on apoptosis in HT-29 colon cancer cells.

In summary, we have shown that kaempferol induces apoptosis in HT29 colon cancer cells containing a mutant *p53* gene. Kaempferol induces changes in the expression and localization of Bcl-2 family proteins: increases Bik; decreases Bcl-xL; decreases intact Bid; and increases mitochondrial Bad. These changes in the levels and localization of Bcl-2 family proteins lead to increases in the permeability of mitochondrial membranes, which subsequently result in cytochrome c release into cytosol. Cytosolic cytochrome c activates caspase-9 and subsequently, it cleaves and activates caspase-3 and caspase-7. These caspases cleave proteins, including PARP, leading to apoptosis. Additionally, kaempferol increases the levels of FAS-L that bind to the FAS receptor, activates caspase-8 that cleaves caspase-3 and Bid. Furthermore, kaempferol induces a reduction in phospho-Akt and Akt activity, which leads to Bad translocation to the mitochondria (Figure 7).

## 3. Experimental Section

### 3.1. Materials

We purchased reagents from the following suppliers: kaempferol (3,4′,5,7-tetrahydroxyflavone), anti-β-actin antibody, essentially fatty acid-free bovine serum albumin (BSA), transferrin, Hoechst 33258, 7-amino-actinomycin D (7-AAD), and 5,5′,6,6′-tetrachloro-1,1′,3,3′-tetraethyl-imidacarbocyanine iodide (JC-1) (Sigma, St. Louis, MO, USA); selenium and DMEM/Ham’s F-12 nutrient mixture (DMEM/F-12; Gibco BRL, Gaithersburg, MD, USA); phycoerythrin conjugated Annexin V (BD Pharmingen, Franklin Lake, NJ, USA); antibodies against Bcl-2, Bcl-xL, Bax, FAS, FAS-L and α-tubulin (Santa Cruz Biotechnology, Santa Cruz, CA, USA); antibodies against cleaved caspase-9, cleaved caspase-3, cleaved caspase-7, cleaved PARP, Bik, Bak, Bim, Bad, Bid, Akt, and phospho-Akt (Cell Signaling, Beverly, MA, USA); antibodies against cytochrome c and caspase-8 (BD Biosciences, San Jose, CA, USA).

### 3.2. Cell Culture

HT-29 cells, SW480 cells, and IEC-6 intestinal cells were purchased from American Type Culture Collection (Manassas, VA, USA) and maintained in DMEM/F12 supplemented with 100 mL/L FBS, 100,000 U/L penicillin and 100 mg/L streptomycin. To examine the effects of kaempferol, cells were plated in 4-well chamber slides (Nunc Labtek, Thermo Fisher Scientific, Waltham, MA, USA), 24-well plates, or 100-mm-diameter dishes. After 24 h, the cell monolayers were serum-starved in DMEM/F12 supplemented with 5 mg/L transferrin, 5 μg/L selenium and 0.1 g/L BSA (serum-free medium) for 24 h. Then, the cells were incubated in a serum-free medium containing various concentrations of kaempferol for the indicated time period.

### 3.3. Hoechst 33258 Staining

Cells were plated in 4-well chamber slides, serum-starved and treated with kaempferol, as described above. Cells were then fixed in 4% paraformaldehyde and stained with a DNA specific dye, Hoechst 33258. Apoptotic cells were observed with a fluorescence microscope (BX51, Olympus, Tokyo, Japan), as previously described [40].

### 3.4. Fluorescence-Activated Cell Sorting (FACS) Analysis

Cells were serum-starved and treated with kaempferol, as described above. To examine whether kaempferol-induced apoptosis is mediated by a caspase-dependent manner, cells were pre-treated with 50 μmol/L Z-VAD-FMK (pan-caspase inhibitor, Sigma, St. Louis, MO, USA) for 1 h. The cells were subsequently treated with 0 or 60 μmol/L kaempferol with or without 50 μmol/L Z-VAD-FMK for 48 h. In order to estimate the apoptotic cell number, cells were trypsinized, loaded with Annexin V and 7-AAD, and then analyzed by flow cytometry (Becton Dickinson, Franklin Lake, NJ, USA), as previously described [34].

### 3.5. Mitochondrial Membrane Potential Indicator Loading Procedure

Cells were serum-starved and treated with kaempferol, as described above. To estimate the mitochondrial membrane potential, cells were trypsinized, loaded with the dual-emission potential-sensitive probe JC-1, and analyzed by flow cytometry, as previously described [40]. JC-1 is a lipophilic membrane-permeant cation that selectively enters the mitochondria and exists in a monomeric form (green fluorescence) or in an aggregated form (red fluorescence) upon mitochondrial hyperpolarization. Therefore, a decrease in JC-1 red/green fluorescence ratio indicates mitochondrial depolarization [41].

### 3.6. Western Blot Analysis and *in Vitro* Kinase Assay

Total cell lysates, cytosolic fractions and mitochondrial fractions were prepared and analyzed by Western blot analysis with their relevant antibodies, as previously described [34,42]. We estimated Akt activity using an Akt kinase assay kit (Cell Signaling, Beverly, MA, USA), as previously described [42]. The relative intensity of each band on immunoblots was quantified by using ImageJ software (NIH, Bethesda, MD, USA). The control levels (0 μmol/L kaempferol) were set at 100%.

### 3.7. Caspase-8 Activity

Caspase-8 activity was measured using ApoAlert Caspase-8 colorimetric assay kit in accordance with the manufacturer’s instructions (Clontech, Mountain View, CA, USA). In brief, the cells were treated with kaempferol for 48 h, and then lysed with Cell Lysis Buffer (supplied with kit). The cell lysates were mixed with the reaction buffer and 200 μmol/L of IETD-pNA (Caspase-8 substrate); they were then incubated for 4 h. The absorbance was measured at 405 nm.

### 3.8. Statistical Analysis

The results were expressed as means ± SEM and were analyzed using the analysis of variance. Differences between groups were assessed by Duncan’s multiple range test, utilizing the SAS statistical software version 9.2 (SAS Institute, Cary, NC, USA).

## 4. Conclusions

Kaempferol treatment induces apoptosis of HT-29 human colon cancer cells through both extrinsic and intrinsic pathways. Kaempferol changes the expression and location of several Bcl-2 family proteins, leading to mitochondrial membrane depolarization and the subsequent release of cytochrome c from the mitochondria. Cytochrome c in the cytosol activates caspase-9, which activates caspase-3. Additionally, kaempferol increases the expression FAS-L, thereby increasing the activity of caspase-8 and ultimately leading to Bid cleavage and caspase-3 activation. Furthermore, kaempferol also activates Akt kinase activity; the increased Akt activity is responsible for Bad translocation to the mitochondria. These observations indicate that the induction of apoptosis is one of the mechanisms by which kaempferol inhibits colon cancer cell growth. DuPont *et al*. [43] reported that the plasma concentrations of kaempferol were 0.1 μmol/L in endive (containing 9 mg kaempferol)-consuming subjects. Additionally, when Sprague-Dawley rats were orally administered with kaempferol (250 mg/kg body weight), the plasma concentrations of kaempferol reach 1.7 μmol/L [44]. These results indicate that consumption of kaempferol-rich foods can increase the plasma concentration of kaempferol. Nevertheless, the plasma concentrations of kaempferol are difficult to reach the concentrations used in our *in vitro* studies. However, because the absorption rate of kaempferol is rather low, the concentration of this flavonol is expected to be much higher in the rumen of the gastrointestinal tract, provided that the consumption of kaempferol is high. Changes in kaempferol levels and its metabolites in the colonic lumen in subjects consuming various amounts of kaempferol should be investigated in the near future.
